# Radiological assessment of the femoral bowing in Japanese population

**DOI:** 10.1051/sicotj/2015037

**Published:** 2016-01-22

**Authors:** Ahmed Hamed Kassem Abdelaal, Norio Yamamoto, Katsuhiro Hayashi, Akihiko Takeuchi, Ahmad Fawaz Morsy, Shinji Miwa, Yoshitomo Kajino, Donnel A. Rubio, Hiroyuki Tsuchiya

**Affiliations:** 1 Department of Orthopedic Surgery, Graduate School of Medical Science, Kanazawa University Kanazawa 9208641 Japan; 2 Department of Orthopedic Surgery, Faculty of Medicine, Sohag University Sohag 82524 Egypt; 3 Department of Orthopedics, Philippine General Hospital, University of the Philippines-Manila Ermita Brgy 670 Zone 72, Manila 1000 Metro Manila Philippines

**Keywords:** Radiological assessment, Femoral bowing, Japanese population

## Abstract

*Introduction*: Differences in the magnitude of bowing between races are well-known characteristics of the femur. Asian races have an increased magnitude of femoral bowing but most of the orthopedic implants designed for the femur do not match this exaggerated bowing. We calculated the sagittal and coronal femoral bowing in the Japanese population at different levels of the femur and addressed its surgical significance.

*Material and methods*: We calculated the sagittal and coronal bowing of 132 Japanese femora using CT scan of the femur. A mathematical calculation of the radius of curvature at proximal, middle, and distal regions of the femur was used to determine the degree of femoral bowing.

*Results*: Mean sagittal bowing of the femur was 581, 188, and 161 mm for the proximal, middle, and distal thirds of the femur and mean lateral bowing was 528, 5092, and 876 mm, respectively. Mean sagittal and coronal bowing for the whole femur was 175 and 2640 mm, respectively. No correlation was found between age, gender, length of femur, and the degree of bowing.

*Conclusion*: Our study reveals that femoral bowing in the Japanese population is 175 mm in the sagittal plane and 2640 mm in the coronal plane; these values are greater than the femoral bowing in other ethnic groups studied in the literature.

This may result in varying degrees of mismatch between the western-manufactured femoral intramedullary implants and the Japanese femur. We recommend that orthopedic surgeons to accurately perform preoperative evaluation of the femoral bowing to avoid potential malalignment, rotation, and abnormal stresses between the femur and implant.

## Introduction

Racial and ethnic group differences in femoral curvature have been studied by anthropologists and forensic scientists [1, 2]. Ballard and Trudell measured anterior femoral curvature by placing femurs on a flat surface and measuring the height of the bow [2]. They found that blacks had a greater radius of curvature than whites. The idea that these racial differences may require modification of orthopedic trauma implants is not a new concept [3].

For decades, trauma and total joint surgeons have struggled to surgically modify the femoral curve to allow the adequate fit and fill of intramedullary nails and stemmed prostheses and anatomically change the inclination angle for the femoral implant guide to avoid possible insertion complications.

Tumor surgery of the lower limb, especially in the femur, requires detailed evaluation of bony anatomy, especially when planning reconstruction with an endoprosthesis. Tumor surgeons must have adequate knowledge and experience in addressing the potentially abnormal bony anatomy which may be encountered in a major reconstructive procedure of the femur.

The osteometry of Asian skeletons differs considerably from that of western populations [4–9]. Bowing of the femur and tibia is not uncommon among Asians. Therefore, accurate alignment and positioning of the intramedullary stem of the tumor endoprosthesis is of paramount importance in achieving stability as well as longevity of the implant. During alignment and orientation of the stem, consideration must be given to the level where the femoral osteotomy is performed and the bowing at that level.

A particular concern during femoral reconstruction with stemmed prostheses after tumor resection is the variable level of osteotomy necessitated by the principles of tumor surgery. The importance of preoperative planning, including assessment of the regional curves of the femur, cannot be overemphasized. The most widely used femoral prostheses for reconstruction are designed according to universal anthropologic measurements and the increased anteroposterior curvature, in addition to coronal curvatures, in the Japanese population is not considered.

In our study, we calculated the measurements for the radii of femoral curvature in both the coronal and sagittal planes in 72 Japanese patients. This is the first study to specifically measure the radius of coronal curvature of the femur in the Japanese population.

## Material and methods

The records of 100 patients who underwent hip surgery from January 2005 to May 2014 at the Department of Orthopedic Surgery in our hospital were reviewed. We excluded patients without full bilateral femur CT scans from the hip to the knee, leaving 72 patients eligible for our study. From 144 femora, 12 were excluded because only postoperative CT scans of the femur were available. A total of 132 femora were included in the study. There were 46 from males and 26 from females with an average age of 47 years ± 14.4 years (range 17–79), they were 65 left and 67 right femora. Fifty-eight were from healthy limbs that did not undergo surgery, and 74 were from limbs that were operated upon, having undergone total hip anthroplasty (THA).

**Figure 1.**
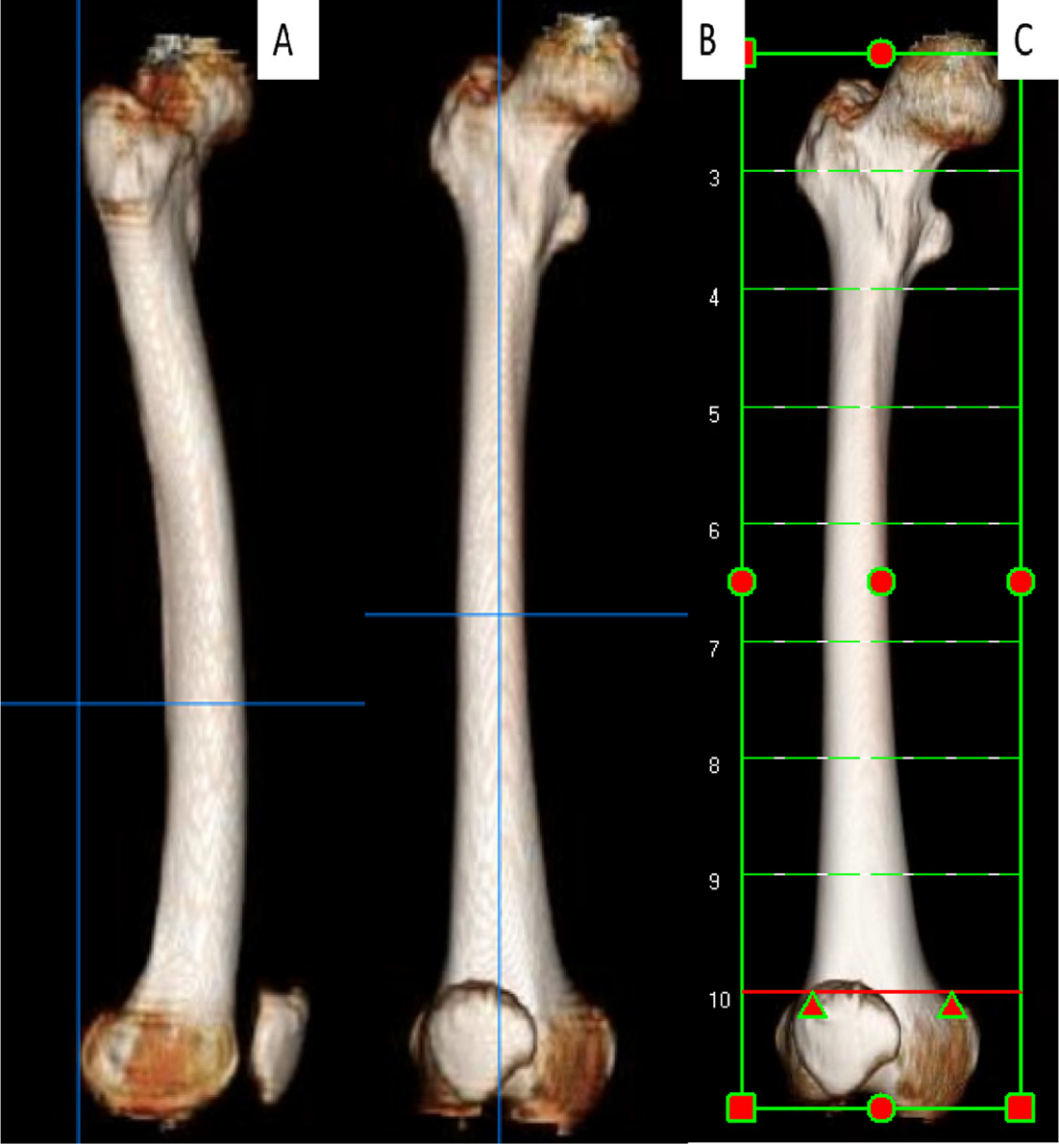
(A) Lateral view of the femur; both condyles are overlapped and a vertical line is tangential to the posterior condylar line and most posterior point of the greater trochanter, (B) anteroposterior view of the femur obtained by rotation of the lateral view 90°, (C) levels of the CT cuts starting at the widest intercondylar diameter and spaced at 5 cm intervals.

**Figure 2.**
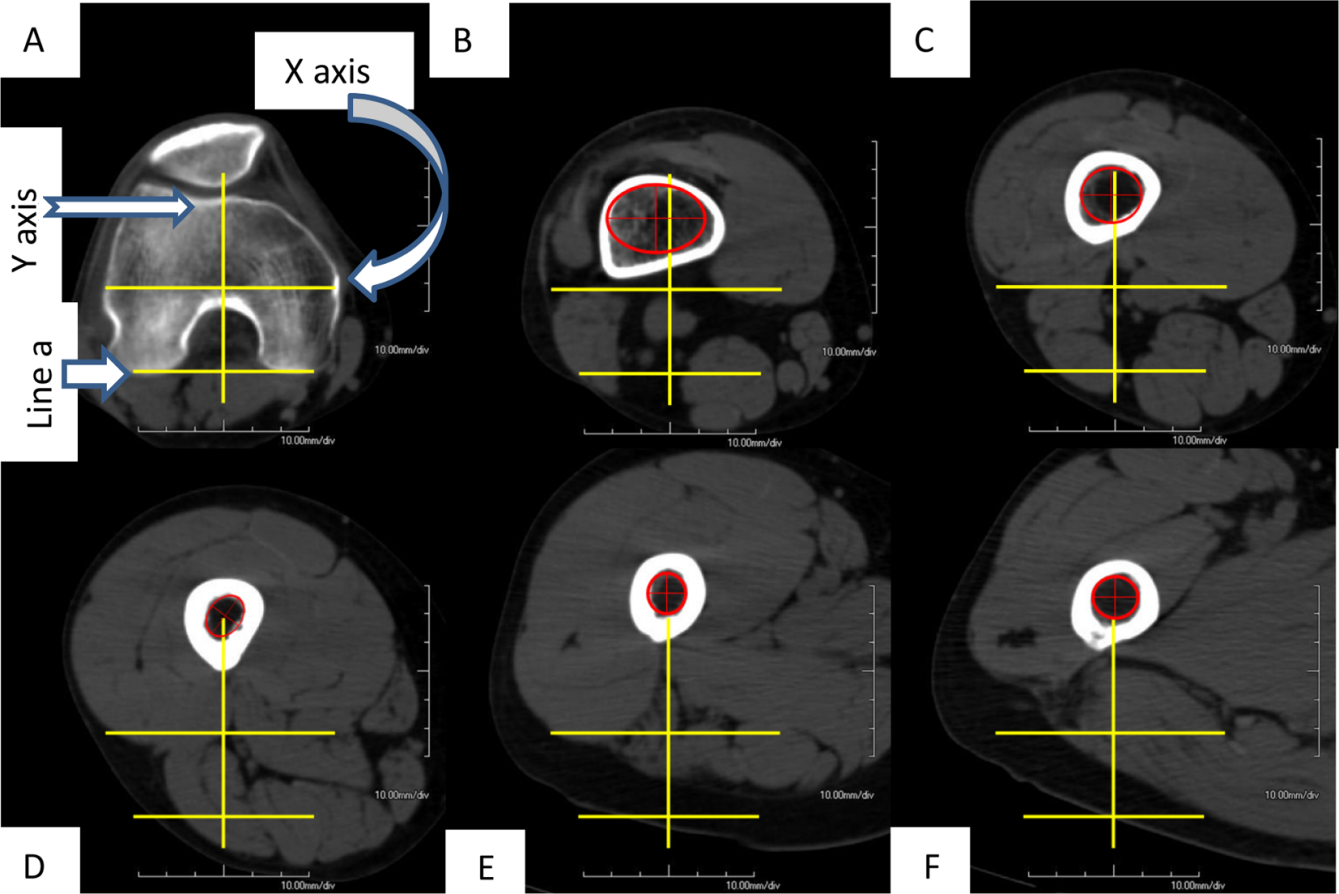
(A) Reference level, intersection between the widest intercondylar diameter and perpendicular line at the middle of both lines. The intersection point is Cartesian or coordinate point (0, 0), (B) center of the medullary canal is represented by the center of the red circle/ellipse best fitting the internal diameter of the medullary canal, (C)–(F) shows the movement of the instantaneous center of the medullary canal along the *Y*-axis (sagittal plane) and *X*-axis (coronal plane).

Using EV Insite version 3.1.1.205 and AquariusNet Viewer V4.8.85 software, we built a 3D model of the femora. For measurements, a direct lateral view was established when a vertical line could be drawn tangential to the most posterior point of both lateral and medial condyles and the most posterior point of the proximal femur (Figure 1A). Using the rotation function, a direct AP view of the femur was achieved by rotating the direct lateral view 90° (Figure 1B). A starting or reference level was chosen at the widest intercondylar point and subsequent 5 cm CT cuts were obtained for each femur (Figure 1C). A total of seven to eight cuts for each femur, depending on its length, were obtained extending from the intercondylar level to the level of lesser trochanter. We determined the central intramedullary point (Figure 2A) by:

1. Drawing a posterior condylar line (a).
2. Drawing a second line (b) at the widest intercondylar distance. This represents the *X*-axis and is parallel to line (a).
3. Drawing a perpendicular to line (b) that bisects the intercondylar notch and represents the *Y*-axis.
4. The point of intersection of the *X* and *Y*-axes is point (0, 0). All cuts are referenced using this coordinate system (Figure 2A).

Using this system for image analysis, the accurate location of the center of the medullary canal, in either AP or lateral planes, can be mathematically depicted as an (*X*, *Y*) value (Figures 2B–2F). The measured *X* values represent the coronal bowing, while *Y* values represent the sagittal bowing of the femur. Readings were measured twice by the first and sixth co-authors, the interobserver reliability was found to be 86%, *κ =* 0.73.

Measurements were then categorized by gender, presence of pathology (±), right or left, and according to length (long femur has eight 5 cm cuts from the widest intercondylar point to the lesser trochanter or short – seven 5 cm cuts).

The radius of curvature of the femur in either coronal or sagittal views at the proximal, middle, and distal thirds of the femur was mathematically calculated using the following formula

$$\mathrm{Radius}\;\left[10\right]=\;\sqrt{{\left(X1-\mathrm{Xc}\right)}^{2}+{\left(Y1-\mathrm{Yc}\right)}^{2}}.$$

*Xc* and *Yc* are the coordinates at the center of the circle/ellipse that represent the measurement of radius of curvature at each level. The radius of curvature was calculated at three levels of the femur: 10, 20, and 30 cm from the intercondylar reference level. We divided the femur into three levels as many arthroplasty surgeries, either THA or TKA, are confronted by such exaggerated femoral bowing in proximal and distal parts of the femur, as well as tumor surgery which may include tumor resection and insertion of a tumor prosthesis for the proximal or distal femur. The alignment and rotation of these implants are directly affected by the degree of femoral bowing in proximal and distal parts, in addition to intramedullary nails which are also affected by the overall bowing, and their insertion is affected by proximal and distal bowing.

The following formulas were used to calculate the coordinates at the center of the circle representing the measurements of radius of curvature:

$$\mathrm{Xc}\;\left[10\right]=\frac{m1m2\left(y1-y3\right)+m2\left(x1+x2\right)-m1\left(x2+x3\right)}{2\left(m2-m1\right)}.$$

$$\;\mathrm{Yc}[10]=-\frac{1}{m1}\left(\mathrm{Xc}-\frac{X1+X2}{2}\right)\;+\frac{Y1+Y2}{2}$$

- For 10 cm level, (*X*1, *Y*1) is at 5 cm level, (*X*2, *Y*2) is at 10 cm level, and (*X*3, *Y*3) is at 15 cm level.
- For 20 cm level, (*X*1, *Y*1) is at 15 cm level, (*X*2, *Y*2) is at 20 cm level, and (*X*3, *Y*3) is at 25 cm level.
- For 30 cm level, (*X*1, *Y*1) is at 25 cm level, (*X*2, *Y*2) is at 30 cm level, and (*X*3, *Y*3) is at 35 cm level.
- M1 is the slope of the line 1 joining point 1 and point 2; it is calculated by this formula:

$$m1\left[10\right]=\frac{x2-x1}{y2-y1}.$$

- M2 is the slope of the line 2 joining point 2 and point 3, it calculated by this formula:

$$m2\left[10\right]=\frac{x3-x2}{y3-y2}.$$

This study is approved by our IRB No. 1730-2 on April 3rd, 2015.

## Results

The center of the medullary canal in 132 Japanese femora was determined and using the (*X*, *Y*) coordinate system, a mathematical model was created to calculate the average femoral bowing in the sagittal and coronal planes.

**Figure 3.**
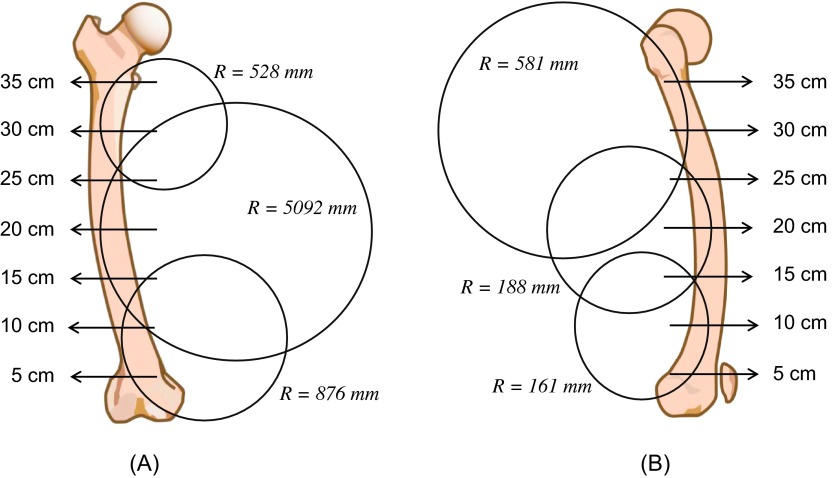
(A) Segmental radii of curvature in the coronal plane of the femur, showing that the Japanese femur is more laterally bowed in proximal, followed by distal and to a much less degree the middle part, (B) segmental radii of curvature in the sagittal plane of the femur, showing that the Japanese femur is more anteroposteriorly bowed in distal, followed by middle and lastly the proximal part.

Mean values for *X* and *Y* coordinates at 5 cm thickness CT intervals were measured in all cases. The mean radius of curvature of the femora in our study was calculated in both the coronal and sagittal planes. Each femur was divided into three regional curves, distal, middle, and proximal, whose centers were considered to be at 10, 20, and 30 cm from the intercondylar reference point, respectively Figures 3A and 3B. The average radii of curvature in the sagittal plane for the three regional curves were found to be 581 mm, 188 mm, and 161 mm for proximal, middle, and distal thirds, respectively. While the average radii of curvature of the coronal plane were found to be 528, 5092, and 876 mm for proximal, middle, and distal thirds, respectively. The average radii of curvature of the whole femur in sagittal and coronal planes were found to be 175 and 2640 mm, respectively Table 1.

**Table 1.** Summarizes our measurements of radii of curvature in sagittal and coronal planes of Japanese femora in proximal, middle, and distal portions as well as the whole femoral bowing.

Anatomical portion	Sagittal radius of curvature (mm)	Coronal radius of curvature (mm)
Whole femur	175	2640
Proximal	581	528
Middle	188	5092
Distal	161	876

All cases were categorized according to gender, presence of pathology, side, and length. There was no statistically significant difference (*using Mann-Whitney test*) between males and females in the mean values for *X* and *Y*, representing coronal and sagittal bowing, respectively (*p* = 0.854). There was no significant difference between right and left femora (*p* = 0.467), between the length of the femur and bowing magnitude (*p* = 0.837), or between the femora of patients who underwent THA surgery and those who did not (*p* = 0.734).

## Discussion

Many studies have addressed the racial differences of femoral curvature, either by forensic investigators who used the femur in racial identifications or by anatomists. Clinical studies focused on the detailed anatomy of the femur and its curvatures and applied this anatomical data in the design of orthopedic implants (i.e. TKA and intramedullary nails). Complications of a mismatch between the conformation of an intramedullary (IM) nail and the bony anatomy of the femur include angular defects [11, 12], iatrogenic fractures [13], and inadequate contact at the fracture line and penetration of the distal anterior femoral cortical bone [3], which may negatively affect fracture healing [14]. Difficulty in removing the mismatched nail [3, 11, 15] or difficulties in inserting the locking screws as a result of deformity due to elongation or bending of the nail are additional problems that can be encountered [3, 15].

Accurate evaluation of femoral bowing is not only essential in total joint arthroplasty and fracture surgery, but it is often more critical for orthopedic tumor surgeons who are frequently challenged by the wide range of variations in femoral bowing. They are often confronted by excessive femoral bowing in either the sagittal or the coronal plane or both. Following the rules of oncological surgery, the tumor extent is assessed and the level of femoral osteotomy individualized. When reconstruction of the limb by stemmed tumor prosthesis is planned, consideration must be given to the variable planes and magnitude of femoral bowing and the possible mismatch which may be encountered when implanting a standard curved stem with unidirectional sagittal plane bowing and no curve in the coronal plane. Therefore, if there is a significantly bowed femur, as prevalent in the Japanese population, it may be necessary to rotate the stem of the implant to accommodate the coronal bowing of the femur using the inherent sagittal curve of the implant. Insertion as such can jeopardize the normal biomechanics of the prosthetic knee joint and future stability of the implant. This is more critical when using cementless implants where stability depends to a great extent on adequate bone contact with no stress shielding as opposed to using cemented implants where the cement, acting as a space filler, can provide adequate canal fill and compensate for the problem of mismatching. The adverse effect at the knee joint if the implant is positioned in rotation is still present, however.

Sagittal plane or anteroposterior curvature is more obvious in the Japanese than that of previous reports of other racial or ethnic groups. In our study, the mean radius of curvature of the femur was found to be 175 mm in the sagittal plane, which is far less than that reported by Marrat et al. who calculated the average medullary radius of curvature to be 1120 mm [16]. In Americans, including different racial groups, African and Asian Americans had much higher values for their anteroposterior femoral bowing, 1222 mm and 1011 mm, respectively. They used CT scan images and software to measure the curvature in a large number of patients. These values are similar to values reported previously by Egol et al. 1200 mm [15] and Harper and Carson 1140 mm [17] who used the cadaveric femora to measure the bowing magnitude.

Harma et al. found the mean medullary bowing to be 722 mm in the Turkish population using radiographs and mathematical calculation [18]. Though this wide variation of measurement is clear, we cannot attribute this wide divergence to ethnic difference alone, otherwise, our deriving will be biased, as each of the previous studies had used a different method, either cadaveric or radiological study, to evaluate the bowing magnitude. Moreover, each of the radiological studies used its unique software program, algorithm, and reference points, due to lack of a standard method for the measurement of femoral bowing. All these factors reduce the assumption that this wide variation is attributed to ethnic diversity alone. This hypothesis is supported by the results of a previous study looking at atypical fractures of the femur (AFF) in Japanese females, where Yoto et al. measured the average medullary bowing in five cases and found it to be 594 mm [19]. They used fluoroscopy to get the lateral view image of the femur and then they measured the radius of curvature using the software. Their results are different from results of the current study, which may also be attributed to difference in measurement methods and number of cases, five versus 132. However, their results still report a more femoral bowing in their patients than previously reported studies.

Regional measurements of the radius of curvature of the femur in the proximal, middle, and distal thirds are not well investigated in the literature, as the main focus has been limited to studying distal femoral anatomy as related to TKA surgeries. In our study, femoral anteroposterior bowing was measured in proximal, middle, and distal thirds and found to be 580 mm, 188 mm, and 161 mm, respectively. These values are much less than what Tang et al. measured in the Chinese population, where the average radii of curvature of the proximal, middle, and distal thirds of the femora were 1081.6 mm, 926.2 mm, and 715.1 mm, respectively [20]. This indicates that the sagittal femoral bow in the Japanese is greater than that of their closest racial group.

**Figure 4.**
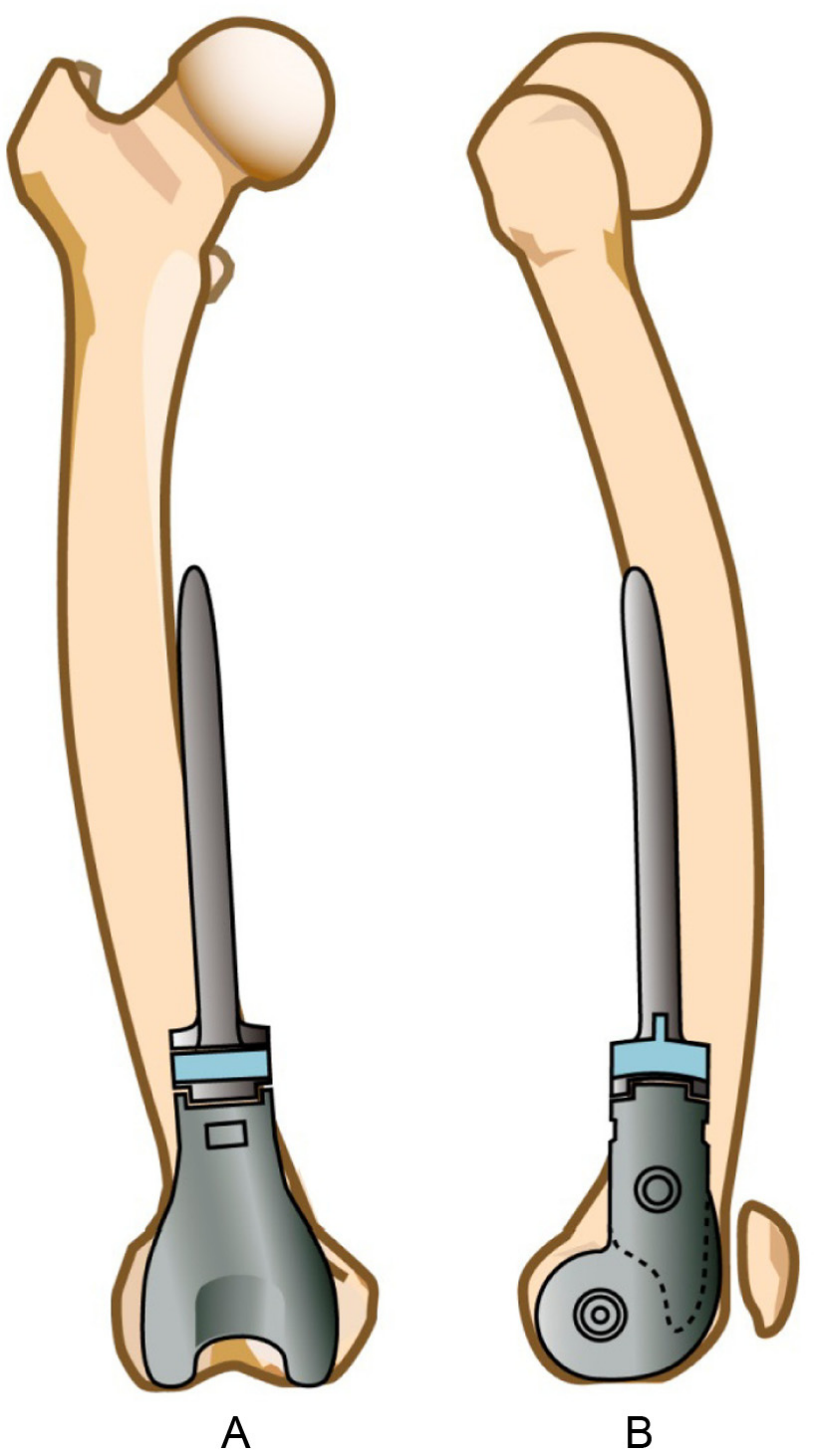
(A) Mismatch between the lateral femoral curve and the stem of the tumor prosthesis, which lacks a coronal plane curve, (B) mismatch between the anteroposterior femoral curve and the stem of a tumor prosthesis having sagittal plane radius of curvature of 900 mm, which is much less than the mean radius of curvature of the Japanese femur.

**Figure 5.**
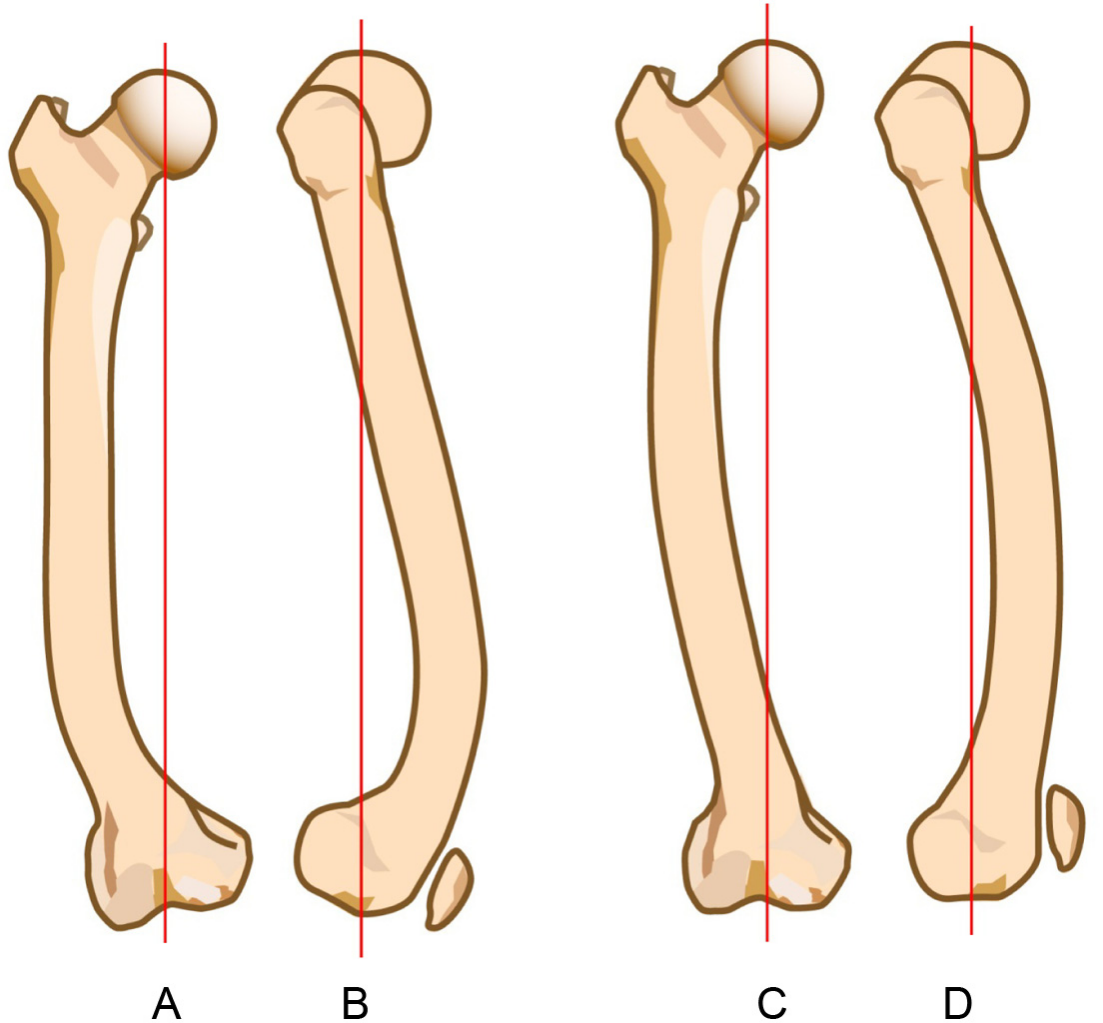
(A) and (B) represents the “J” shape of Chinese femoral curves in anteroposterior and lateral views, respectively, (C) and (D) represents the “parenthesis” shape of Japanese femoral curves in anteroposterior and lateral views, respectively.

Coronal plane bowing of the femur is another characteristic documented in the Japanese and Chinese people [21, 22]. Arthroplasty and trauma surgeons who fail to recognize this characteristic preoperatively and appropriately adjust the femoral cut inclination angle or entry point of the IM are often beset by unusual intraoperative complications or marginal results as noted on postoperative X-rays. Tumor surgeons also are often challenged to adapt the uniplanar curved tumor prosthesis into a biplanar position in the medullary canal. A narrow canal may prompt the surgeon to ream excessively and remove significant amounts of bone stock or force the surgeon to rotate the implant to fit the coronal bow of the femur, which in turn affects the knee joint mechanics and accelerates implant loosening postoperatively (Figures 4A and 4B). In our cases, lateral bowing of the femur was characterized by the radius of curvature and was measured to be 2640 mm over the entire length of the femur. Segmental measurements for lateral bowing revealed that the radii of curvature for the proximal, middle, and distal thirds were 528 mm, 5092 mm, and 876 mm, respectively, demonstrating the fact that the femur was curved to a great degree in its proximal segment, followed by the distal portion, and to a much less extent the middle segment. Descriptively it resembles a “parenthesis mark” (Figure 5).

Tang et al. described the Chinese femur as “J” shaped as the average radii of curvature for the lateral femoral bowing were 1099.0 mm, 935.2 mm, and 722.3 mm for the proximal, middle, and distal segments, respectively. This indicates that the Chinese femur is bowed the greatest in its distal portion, followed by the middle, and then the proximal portion (Figures 5A–5D) [21]. The radii of curvature from different studies, methods used for measurement, and number of cases are summarized in Table 2.

**Table 2:** Summarizing the results of currently available studies in the literature about femoral curvatures in different races.

Author	Year	Number of cases	Primary method used for measurement	Race/country	Sagittal radius of curvature (mm)	Coronal radius of curvature (mm)
Egol et al. [15]	2004	892	Cadaveric femora	American	1200	
Maratt et al. [16]	2014	3922	CT scan and software	American	1120	
				African	1222	
				Asian	1011	
Harper and Carson [17]	1987	14	Cadaveric femora	American	1140	
Harma et al. [18]	2005	104	Radiographs and mathematical calculation	Turkish	722	
Yoto et al. [19]	2014	5	Radiographs and software	Japanese	594	
Tang et al. [20]	2004	100	Digital radiographs and software	Chinese		
				Proximal	1081	1099
				Middle	926	935
				Distal	715	722

No statistically significant correlation could be proven between the magnitude of the curve and age, gender, side, or length of the femur. Additionally, there was no statistically significant difference between the femora of patients who underwent THA surgery and those who did not. This is partially consistent with what Marrat et al. [16] showed in his study on Americans from different ethnic groups (Asian, African others). He found a moderately strong positive correlation between femoral length and radius of curvature. There was also a moderately strong positive correlation between body stature and radius of curvature. Furthermore, he stated that “each additional centimeter increase in femoral length correlates to a 3.1-cm (right) to 3.4-cm (left) increase in the radius of curvature”. They could not, however, correlate gender, ethnicity, age, BMI, and cortical thickness with femoral length or radius of curvature. Tang et al. stated that shorter femora were correlated with a shorter radius of curvature and although they could not show any gender-related differences, they did find a poor correlation with age (*r* = 0.13) [20].

Zuber et al. [12] found no correlation between the femoral radius of curvature and age, femoral length, or gender. The finding of Maratt et al. [16] and Ballard and Trudell [2] confirmed that blacks had a significantly greater average radius of femoral curvature (i.e. straighter) than whites. They also found males had a greater radius of curvature than females, although this difference was not significant. White males, however, had a significantly greater radius of curvature than white females while black males and black females had similar radii of curvature. In their study, Egol et al. [15] demonstrated no correlation between age and radius of curvature or between femoral width or length and radius of curvature.

By measuring the radius of curvature of the femoral bowing preoperatively in both the sagittal and coronal planes, especially in the proximal and distal thirds of the femur, the necessary contours needed in the tumor prosthesis stem to obtain accurate and matched intramedullary fixation based on the level of the planned femoral osteotomy can be determined. In summary, average measurements of the radius of curvature for sagittal plane bowing in Japanese population seem to be smaller than those of other populations studied in the literature implying a greater degree of anteroposterior bowing of Japanese femur. This characteristic often causes mismatch between the western-oriented and some Japanese-oriented conformations for intramedullary nails, tumor prostheses, and stemmed forms of TKA implants. Japanese femur also has a lateral bowing, more in the proximal femur, followed by distal region and to a much less extent, the midshaft region.

## Conclusion

Our study reveals that femoral bowing in Japanese population is 175 mm in sagittal plane radius of curvature of the femur and 2640 mm in coronal plane radius of curvature of the femur; these values are greater than the femoral bowing in other ethnic groups studied in the literature.

This may result in varying degrees of mismatch between the western-manufactured femoral intramedullary implants, tumor prostheses, and intramedullary types of TKA implants and the Japanese femur. We recommend orthopedic surgeons to accurately perform preoperative evaluation of the femoral bowing to avoid potential malalignment, rotation, and abnormal stresses between the femur and implant.

## Conflict of interest

The authors declare no conflict of interest in relation with this paper.
